# Supplementary material for: Effect of fragmentation on the Costa Rican dry forest avifauna
Source: PeerJ. 2016 Sep 13;4:e2422. doi: 10.7717/peerj.2422 (PMC5028763; doi:10.7717/peerj.2422)
Supplement: Table S1 [file peerj-04-2422-s001.docx]

| Supplemental Table S1. Checklist of resident bird species recorded in five dry forest fragments in northwestern Costa Rica: SR – Santa Rosa, PV – Palo Verde, RV – Rincón de la Vieja, Dir – Diriá, CB – Cabo Blanco. | | | | | | | |
| --- | --- | --- | --- | --- | --- | --- | --- |
| **Family** | **Scientific Name** | **SR** | **PV** | **RV** | **Dir** | **CB** | **Forest Dependency** |
| Tinamidae | *Crypturellus soui* |  |  | x | x | x | 3 |
|  | *Crypturellus cinnamomeus* | x | x | x | x | x | 2 |
| Cracidae | *Ortalis vetula* | x | x |  | x |  | 1 |
|  | *Ortalis cinereiceps* |  |  |  | x | x | 2 |
|  | *Penelope purpurascens* | x | x | x |  | x | 2 |
|  | *Crax rubra* | x | x | x |  | x | 1 |
|  | *Colinus cristatus* | x | x |  | x | x | 3 |
| Cathartidae | *Coragyps atratus* | x | x | x | x | x | 3 |
|  | *Cathartes aura* | x | x | x | x | x | 3 |
|  | *Sarcoramphus papa* | x | x | x | x | x | 2 |
| Accipitridae | *Leptodon cayanensis* |  | x |  | x | x | 1 |
|  | *Chondrohierax uncinatus* | x | x |  |  | x | 1 |
|  | *Harpagus bidentatus* |  | x |  |  |  | 1 |
|  | *Ictinia plúmbea* |  | x |  |  |  | 2 |
|  | *Geranospiza caerulescens* |  | x |  |  |  | 1 |
|  | *Morphnarchus princeps* |  |  | x |  |  | 1 |
|  | *Pseudastur albicollis* |  |  |  |  | x | 2 |
|  | *Buteogallus anthracinus* | x | x |  |  |  | 2 |
|  | *Buteogallus urubitinga* | x | x |  |  |  | 1 |
|  | *Parabuteo unicinctus* |  | x |  |  |  | 2 |
|  | *Buteo magnirostris* | x | x | x | x | x | 3 |
|  | *Buteo nitidus* | x | x | x | x | x | 2 |
|  | *Buteo brachyurus* |  | x |  | x |  | 1 |
|  | *Buteo albicaudatus* | x | x |  |  |  | 3 |
|  | *Buteo albonotatus* | x | x |  | x |  | 2 |
|  | *Micrastur semitorquatus* | x | x | x | x | x | 1 |
|  | *Ibycter americanus* |  |  |  | x |  | 1 |
|  | *Caracara cheriway* | x | x | x | x | x | 3 |
|  | *Milvago chimachima* |  | x |  | x |  | 3 |
|  | *Herpetotheres cachinnans* | x | x | x | x | x | 2 |
| Rallidae | *Aramides cajanea* | x | x |  | x | x | 3 |
| Burhinidae | *Burhinus bistriatus* | x | x |  | x |  | 3 |
| Columbidae | *Patagioenas flavirostris* | x | x | x | x | x | 3 |
|  | *Patagioenas fasciata* | x |  | x |  |  | 2 |
|  | *Patagioenas subvinacea* |  |  |  | x |  | 2 |
|  | *Zenaida asiatica* | x | x | x | x | x | 3 |
|  | *Zenaida macroura* |  | x |  |  |  | 2 |
|  | *Columbina inca* | x | x | x | x | x | 3 |
|  | *Columbina passerina* | x | x | x | x | x | 3 |
|  | *Columbina minuta* |  | x |  | x |  | 3 |
|  | *Columbina talpacoti* |  | x |  | x | x | 3 |
|  | *Claravis pretiosa* | x | x | x | x | x | 3 |
|  | *Leptotila verreauxi* | x | x | x | x | x | 2 |
|  | *Leptotila plumbeiceps* |  |  |  |  | x | 2 |
|  | *Geotrygon costaricensis* |  |  | x |  |  | 1 |
|  | *Geotrygon montana* |  |  | x |  |  | 1 |
| Psittacidae | *Aratinga canicularis* | x | x | x | x | x | 2 |
|  | *Ara macao* | x | x |  |  |  | 2 |
|  | *Brotogeris jugularis* | x | x | x | x | x | 3 |
|  | *Pyrilia haematotis* |  |  | x |  |  | 1 |
|  | *Amazona albifrons* | x | x | x | x | x | 2 |
|  | *Amazona autumnalis* | x |  |  |  |  | 2 |
|  | *Amazona auropalliata* | x | x |  |  |  | 2 |
| Cuculidae | *Piaya cayana* | x | x | x | x | x | 2 |
|  | *Tapera naevia* |  | x |  |  | x | 3 |
|  | *Morococcyx erythropygius* | x | x | x | x | x | 2 |
|  | *Crotophaga sulcirostris* | x | x | x | x | x | 3 |
| Tytonidae | *Tyto alba* | x | x | x |  | x | 2 |
| Strigidae | *Megascops cooperi* | x | x | x | x | x | 2 |
|  | *Megascops guatemalae* |  |  |  | x |  | 1 |
|  | *Pulsatrix perspicillata* | x | x |  | x | x | 2 |
|  | *Glaucidium brasilianum* | x | x | x | x | x | 2 |
|  | *Ciccaba virgata* | x | x | x | x |  | 2 |
|  | *Pseudoscops clamator* |  | x |  |  |  | 3 |
| Caprimulgidae | *Chordeiles acutipennis* | x | x | x |  | x | 3 |
|  | *Chordeiles minor* | x | x |  |  |  | 3 |
|  | *Nyctidromus albicollis* | x | x | x | x | x | 3 |
|  | *Nyctibius jamaicensis* | x | x |  |  |  | 2 |
| Trochilidae | *Phaethornis striigularis* | x | x | x | x | x | 2 |
|  | *Phaeochroa cuvierii* |  | x | x |  | x | 2 |
|  | *Anthracothorax prevostii* | x | x | x | x | x | 3 |
|  | *Chlorostilbon canivetii* | x | x | x | x | x | 3 |
|  | *Panterpe insignis* |  |  | x |  |  | 2 |
|  | *Hylocharis eliciae* | x | x | x | x | x | 2 |
|  | *Amazilia saucerrottei* | x | x | x | x | x | 2 |
|  | *Amazilia tzacatl* | x | x | x | x | x | 3 |
|  | *Amazilia rutila* | x | x | x | x | x | 2 |
|  | *Lampornis castaneoventris* |  |  | x |  |  | 2 |
|  | *Heliomaster constantii* | x | x |  | x |  | 2 |
| Trogonidae | *Trogon massena* |  |  | x |  |  | 2 |
|  | *Trogon melanocephalus* | x | x | x | x | x | 2 |
|  | *Trogon caligatus* | x | x | x | x | x | 2 |
|  | *Trogon elegans* | x | x | x | x |  | 1 |
| Momotidae | *Momotus momota* | x | x | x | x | x | 2 |
|  | *Electron carinatum* |  |  | x |  |  | 2 |
|  | *Eumomota superciliosa* | x | x | x | x | x | 3 |
| Ramphastidae | *Aulacorhynchus prasinus* |  |  | x |  |  | 2 |
|  | *Pteroglossus torquatus* | x | x | x | x | x | 2 |
|  | *Selenidera spectabilis* |  |  | x |  |  | 2 |
|  | *Ramphastos sulfuratus* |  |  | x |  |  | 1 |
|  | *Ramphastos ambiguus* |  |  |  | x |  | 1 |
| Picidae | *Melanerpes hoffmannii* | x | x | x | x | x | 2 |
|  | *Dryocopus lineatus* | x | x | x | x | x | 2 |
|  | *Campephilus guatemalensis* | x | x | x | x | x | 2 |
| Furnariidae | *Sclerurus albigularis* |  |  | x |  |  | 1 |
|  | *Dendrocincla homochroa* | x | x | x | x | x | 1 |
|  | *Sittasomus griseicapillus* | x | x | x | x | x | 2 |
|  | *Dendrocolaptes sanctithomae* | x | x | x | x | x | 2 |
|  | *Xiphorhynchus flavigaster* | x | x |  |  | x | 2 |
|  | *Lepidocolaptes souleyetii* | x | x | x | x | x | 2 |
| Thamnophilidae | *Thamnophilus doliatus* | x | x | x | x | x | 2 |
|  | *Dysithamnus mentalis* |  |  | x |  |  | 1 |
|  | *Hylophylax naevioides* |  |  | x |  |  | 2 |
| Tyrannidae | *Camptostoma imberbe* | x | x | x |  |  | 2 |
|  | *Myiopagis viridicata* | x | x | x |  |  | 2 |
|  | *Elaenia flavogaster* | x | x |  |  |  | 3 |
|  | *Elaenia frantzii* |  |  | x |  |  | 2 |
|  | *Mionectes oleagineus* | x |  |  |  | x | 2 |
|  | *Zimmerius vilissimus* |  | x |  |  | x | 2 |
|  | *Oncostoma cinereigulare* |  |  |  |  | x | 2 |
|  | *Poecilotriccus sylvia* | x | x |  |  |  | 2 |
|  | *Todirostrum cinereum* | x | x | x | x | x | 2 |
|  | *Tolmomyias sulphurescens* | x | x |  | x | x | 3 |
|  | *Tolmomyias assimilis* |  |  | x |  |  | 1 |
|  | *Platyrinchus cancrominus* |  |  | x |  | x | 1 |
|  | *Onychorhynchus coronatus* | x |  |  | x |  | 1 |
|  | *Myiobius sulphureipygius* |  |  | x |  |  | 1 |
|  | *Contopus cinereus* | x |  | x |  |  | 3 |
|  | *Sayornis nigricans* | x |  | x |  |  | 3 |
|  | *Attila spadiceus* | x | x | x | x | x | 2 |
|  | *Myiarchus tuberculifer* | x | x | x | x |  | 2 |
|  | *Myiarchus nuttingi* | x | x |  |  |  | 2 |
|  | *Myiarchus tyrannulus* | x | x | x | x | x | 2 |
|  | *Pitangus sulphuratus* | x | x | x | x | x | 3 |
|  | *Megarynchus pitangua* | x | x | x | x | x | 2 |
|  | *Myiozetetes similis* | x | x | x | x | x | 3 |
|  | *Myiodynastes maculatus* | x | x |  | x | x | 2 |
|  | *Tyrannus melancholicus* | x | x | x |  | x | 3 |
| Tityridae | *Pachyramphus cinnamomeus* | x | x |  |  |  | 2 |
|  | *Pachyramphus polychopterus* | x | x |  | x |  | 2 |
|  | *Pachyramphus aglaiae* | x | x | x | x | x | 2 |
|  | *Tityra semifasciata* | x | x | x | x | x | 2 |
|  | *Tityra inquisitor* | x | x |  |  |  | 2 |
| Pipridae | *Chiroxiphia linearis* | x | x | x | x | x | 2 |
| Vireonidae | *Hylophilus ochraceiceps* |  |  | x |  |  | 2 |
|  | *Hylophilus decurtatus* | x | x | x | x | x | 2 |
|  | *Cyclarhis gujanensis* | x | x | x | x | x | 2 |
| Corvidae | *Calocitta formosa* | x | x | x | x | x | 2 |
|  | *Psilorhinus morio* | x | x | x |  |  | 3 |
|  | *Tachycineta albilinea* | x | x |  |  |  | 3 |
|  | *Pygochelidon cyanoleuca* |  |  | x |  |  | 3 |
|  | *Stelgidopteryx ruficollis* |  | x | x |  |  | 3 |
| Troglodytidae | *Campylorhynchus rufinucha* | x | x | x | x | x | 2 |
|  | *Thryophilus rufalbus* | x | x | x | x | x | 1 |
|  | *Thryophilus pleurostictus* | x | x | x | x | x | 2 |
|  | *Cantorchilus modestus* | x | x | x | x | x | 3 |
|  | *Troglodytes aedon* | x | x | x |  | x | 3 |
|  | *Henicorhina leucophrys* |  |  | x |  |  | 1 |
| Polioptilidae | *Ramphocaenus melanurus* | x | x |  | x | x | 2 |
|  | *Polioptila albiloris* | x | x |  | x |  | 2 |
|  | *Polioptila plumbea* | x | x | x | x | x | 2 |
| Turdidae | *Myadestes melanops* |  |  | x |  |  | 1 |
|  | *Catharus aurantiirostris* |  |  |  | x |  | 2 |
|  | *Catharus mexicanus* |  |  | x |  |  | 1 |
|  | *Turdus plebejus* |  |  | x |  |  | 2 |
|  | *Turdus grayi* | x | x | x | x | x | 3 |
|  | *Turdus assimilis* |  |  | x |  |  | 2 |
| Ptilogonatidae | *Phainoptila melanoxantha* |  |  | x |  |  | 2 |
| Parulidae | *Geothlypis poliocephala* | x | x | x | x | x | 3 |
|  | *Myioborus miniatus* |  |  | x |  |  | 2 |
|  | *Basileuterus culicivorus* |  |  | x |  |  | 1 |
|  | *Basileuterus rufifrons* | x | x | x | x | x | 2 |
| Incertae sedis | *Coereba flaveola* |  |  |  |  | x | 2 |
| Thraupidae | *Eucometis penicillata* |  | x | x | x | x | 1 |
|  | *Thraupis episcopus* | x | x | x | x | x | 2 |
|  | *Thraupis palmarum* | x | x | x | x | x | 2 |
|  | *Dacnis cayana* |  |  | x |  |  | 2 |
|  | *Cyanerpes cyaneus* |  | x | x | x | x | 2 |
| Emberizidae | *Volatinia jacarina* | x | x |  | x | x | 3 |
|  | *Sporophila torqueola* | x | x | x | x | x | 3 |
|  | *Tiaris olivaceus* |  |  |  | x |  | 3 |
|  | *Diglossa plumbea* |  |  | x |  |  | 2 |
|  | *Arremonops rufivirgatus* | x | x | x | x | x | 1 |
|  | *Peucaea ruficauda* | x | x | x | x | x | 2 |
|  | *Chlorospingus ophthalmicus* |  |  | x |  |  | 2 |
| Cardinalidae | *Habia rubica* |  |  | x | x | x | 1 |
|  | *Passerina caerulea* | x | x | x | x |  | 3 |
| Icteridae | *Sturnella magna* |  | x | x |  |  | 3 |
|  | *Dives dives* | x | x |  | x |  | 3 |
|  | *Quiscalus mexicanus* | x | x | x | x | x | 3 |
|  | *Molothrus aeneus* | x | x |  |  |  | 3 |
|  | *Icterus pustulatus* | x | x | x | x |  | 3 |
|  | *Icterus pectoralis* | x | x |  |  |  | 2 |
|  | *Amblycercus holosericeus* |  |  |  | x |  | 3 |
|  | *Psarocolius montezuma* |  | x | x |  | x | 2 |
| Fringillidae | *Euphonia affinis* | x | x |  | x | x | 2 |
|  | *Euphonia hirundinacea* | x |  | x | x | x | 2 |
|  |  |  |  |  |  |  |  |
| Total species | 187 | 123 | 135 | 127 | 109 | 104 |  |
